# Changes in Self‐Perceived Competence Following a Virtual Workshop on Cognitive Assessment

**DOI:** 10.1002/alz70858_102891

**Published:** 2025-12-25

**Authors:** David Cowan, Jennifer Siemon, Anjali Sarkar, Rachel Plagos, Emily Speelziek, Katie Harder, Susie Gregg

**Affiliations:** ^1^ McMaster University, Hamilton, ON, Canada; ^2^ Hamilton Health Sciences, Hamilton, ON, Canada; ^3^ Cambridge Memorial Hospital, Cambridge, ON, Canada; ^4^ Providence Healthcare, Toronto, ON, Canada; ^5^ Grand River Hospital, Kitchener, ON, Canada; ^6^ Canadian Mental Health Association Waterloo Wellington, Guelph, ON, Canada

## Abstract

**Background:**

Cognitive assessment is a necessary part of diagnosing Alzheimer's Disease and related disorders, monitoring disease progression, and measuring treatment effectiveness. Geriatricians and other healthcare providers have observed that cognitive tests are often not administered in a standardized manner, compromising their validity. As well, they are often not administered on the appropriate population or in the appropriate setting. The Cognitive Assessment Tools (CAT) workshop is a half‐day, interactive, small group program delivered virtually by trained and experienced healthcare providers. The CAT workshop equips participants with knowledge on the appropriate usage, interpretation, and standardized administration and scoring of cognitive assessment tools, including the Standardized Mini‐Mental Status Exam (sMMSE), Montreal Cognitive Assessment (MoCA®), Trail Making Test (TMT), Clock Drawing Test (CDT), and Geriatric Depression Scale (GDS). This workshop was previously offered in person, in both small‐ and large‐group formats. The present study aims to evaluate outcomes of this novel workshop delivered in a virtual format.

**Method:**

Workshop participants completed pre‐ post‐workshop surveys. Participants utilized a scale from 1 (no proficiency) to 7 (mastery) to report their self‐perceived competency.

**Result:**

53 participants from 9 workshops completed pre‐ and post‐ workshop evaluations (57% response rate). Participants’ professions included: nursing (56%), social work (9%), occupational therapy (6%), therapeutic recreation (6%), medicine (4%), physiotherapy (4%), personal support worker (4%), and other (11%). Results of paired t‐tests indicated a significant difference between before and after scores for all cognitive assessments. Pre‐post mean differences for each cognitive test: sMMSE=1.81; MoCA®=1.70; TMT=2.47; CDT=1.75; GDS=2.11, all *p* <0.001. There was a significant difference in participants’ understanding of why cognitive assessments are used (pre‐post mean difference = 1.15), and their knowledge of selecting the appropriate tool to use (pre‐post mean difference = 2.21), both *p* <0.001. Ninety‐four percent of participants would recommend the workshop to a colleague.

**Conclusion:**

The CAT workshop is associated with significant increases in healthcare workers’ self‐reported competency in selecting, administering, scoring and interpreting common cognitive assessment tools. The workshop attracts a variety of healthcare and social care professionals, and is well‐received by learners.

